# A high-resolution genome of an euryhaline and eurythermal rhinogoby (*Rhinogobius similis* Gill 1895)

**DOI:** 10.1093/g3journal/jkab395

**Published:** 2021-11-18

**Authors:** Yun Hu, Liang Lu, Tao Zhou, Kishor Kumar Sarker, Junman Huang, Jianhong Xia, Chenhong Li

**Affiliations:** 1 Shanghai Universities Key Laboratory of Marine Animal Taxonomy and Evolution, Shanghai Ocean University, Shanghai 201306, China; 2 Shanghai Collaborative Innovation for Aquatic Animal Genetics and Breeding, Shanghai Ocean University, Shanghai 201306, China; 3 Shanghai Natural History Museum, Branch of the Shanghai Science & Technology Museum, Shanghai 200041, China

**Keywords:** *Rhinogobius similis*, nanopore sequencing, Hi-C (high-throughput chromosome conformation capture), PSMC (pairwise sequentially Markovian coalescent analysis), freshwater goby, transcriptome

## Abstract

*Rhinogobius similis* is distributed in East and Southeast Asia. It is an amphidromous species found mostly in freshwater and sometimes brackish waters. We have obtained a high-resolution assembly of the *R. similis* genome using nanopore sequencing, high-throughput chromosome conformation capture (Hi-C), and transcriptomic data. The assembled genome was 890.10 Mb in size and 40.15% in GC content. Including 1373 contigs with contig N50 is 1.54 Mb, and scaffold N50 is 41.51 Mb. All of the 1373 contigs were anchored on 22 pairs of chromosomes. The BUSCO evaluation score was 93.02% indicating high quality of genome assembly. The repeat sequences accounted for 34.92% of the whole genome, with retroelements (30.13%), DNA transposons (1.64%), simple repeats (2.34%), and so forth. A total of 31,089 protein-coding genes were predicted in the genome and functionally annotated using Maker, of those genes, 26,893 (86.50%) were found in InterProScan5. There were 1910 gene families expanded in *R. similis*, 1171 gene families contracted and 170 gene families rapidly evolving. We have compared one rapidly change gene family (*PF05970*) commonly found in four species (*Boleophthalmus pectinirostris*, *Neogobius melanostomus*, *Periophthalmus magnuspinnatus*, and *R. similis*), which was found probably related to the lifespan of those species. During 400–10 Ka, the period of the Guxiang Ice Age, the population of *R. similis* decreased drastically, and then increased gradually following the last interglacial period. A high-resolution genome of *R. similis* should be useful to study taxonomy, biogeography, comparative genomics, and adaptive evolution of the most speciose freshwater goby genus, *Rhinogobius*.

## Introduction

The genus, *Rhinogobius* Gill, 1859 is the most speciose freshwater goby distributed in the drainages that flow into the coast of Western Pacific. Due to its high endemism, there are many species found in this genus, including 85 species mentioned in Yamasaki *et al.* (2015) and 15 newly described ones thereafter. Furthermore, there may exist many more cryptic species (Wu *et al.* 2016). Because many meristic characters are overlapped between different species and most diagnostics are based on coloration patterns (Ohara *et al.* 2004; Cheng *et al.* 2005), the phylogeny of *Rhinogobius* is largely unresolved, with only local keys proposed and phylogeny of species in restricted regions studied (Chen and Shao 1996; Chen *et al.* 2002; Wu 2008; Yamasaki *et al.* 2015).

The species of *Rhinogobius* can adapt to various salinity and temperature. There are both amphidromous species tolerant to wide salinity range and landlocked ones that are strictly freshwater in this genus (Takahashi and Yanagisawa 1999; Li and Zhong 2009). They also can bear different temperature variation, with some species have wide distribution latitudinally and tolerate a wide temperature ranges; instead, some are stenothermal living in spring-fed creeks (Li and Zhong 2009; Ju *et al.* 2016). Therefore, the *Rhinogobius* also could be a good model to study molecular mechanisms underlying adaptation to salinity and temperature variation in bony fishes. Finally, the *Rhinogobius* could be a good group for investigating molecular mechanisms underlying morphological variation, such as color patterns. For example, some species have stripes on their cheek, whereas other closely related ones have specks (Zheng 1985; Zhong and Tzeng 1998).

Suzuki *et al.* (2016) re-described *Rhinogobiussimilis*, the type species of the genus *Rhinogobius* Gill 1859 and designated *R. giurinus* as its synonymy. *Rhinogobius similis* is a euryhaline and eurythermal species, having the widest distribution among the species of this genus (Rutter 1897; Kim *et al.* 1997; Tzeng *et al.* 2006). *Rhinogobius similis* also is the earliest branch splitting off the tree of this genus (Yamasaki *et al.* 2015). Assembling a high-resolution genome of *R. similis*, the first genome of the genus, would provide important resources for studying taxonomy, biogeography, adaptation, and answer many other interesting questions in this fascinating group of fishes.

## Materials and methods

### Sample collection

**Figure 1:**
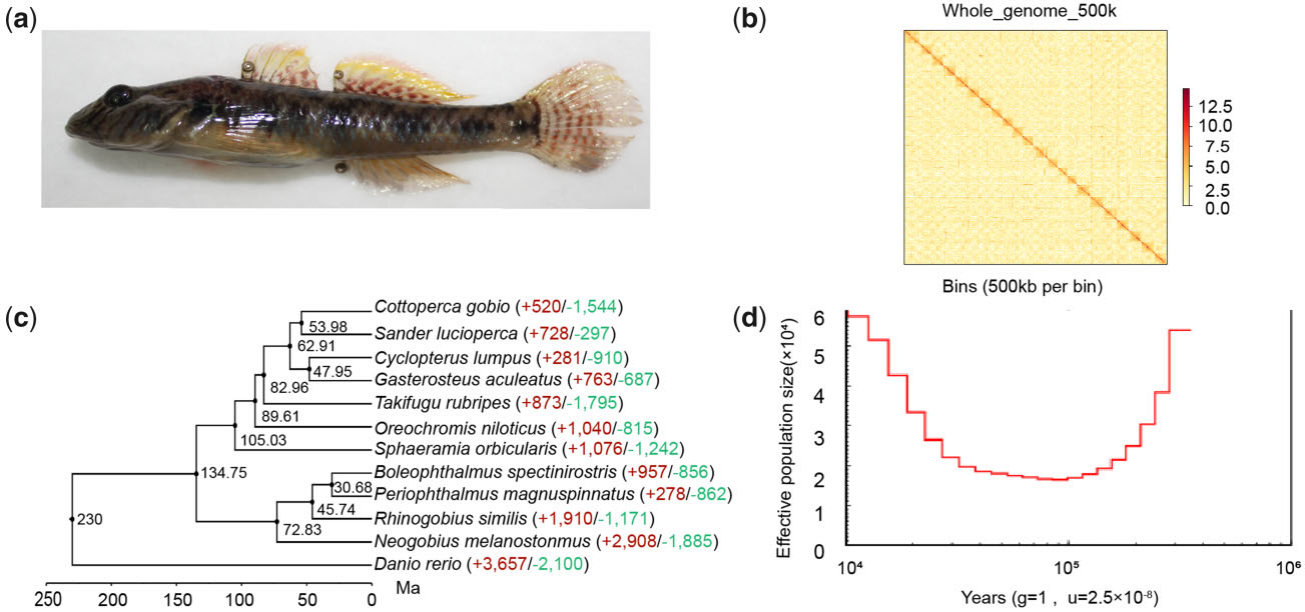
(A) The specimen of *Rhinogobius similis* collected on campus of Shanghai Ocean University; (B) Hi-C interaction heat map, the yellow rectangle represents 22 scaffolds. The color block depth of each pixel point represents the number of contacts of chromatin DNA after homogenization; (C) the time tree of *R. similis*. The “+” sign indicates the number of expanded gene families, whereas “–” sign indicates the number of contracted gene families; (D) The historical population size of *R. similis* from 400 to 10 Ka estimated using pairwise sequentially Markovian coalescent (PSMC).

A male *R. similis* was collected from the lake on campus of the Shanghai Ocean University, Shanghai, China (30°53′1.7777″N, 121°53′40.8768″E) on November 15, 2020 (Figure 1). The total length of this specimen is 67.24 mm.

### DNA preparation, nanopore sequencing, data generating for assembly polishing, and chromosome-scale scaffolding

Genomic DNA was extracted from two muscle samples using CTAB method according to QIAGEN^®^ Genomic kit (Cat#13343, QIAGEN). About 3–4 µg DNA was taken for constructing DNA library and sequenced on a Nanopore PromethION sequencer instrument (Oxford Nanopore Technologies, UK) at Genome Center of GrandOmics (Wuhan, China). An Illumina sequencing library was constructed using the modified library preparation method using DNA sheared to ∼250 bp (Meyer and Kircher 2010; Li *et al.* 2013). The library was sequenced using an Illumina HiSeq platform at GENEWIZ (Suzhou, Jiangsu, China). The sequence data were used to polish the assembly from the nanopore sequencing results. The Hi-C library construction and sequencing were performed at GrandOmics following the pipeline of Belton *et al.* (2012).

### RNA library construction and sequencing

The RNA library construction and sequencing were performed at GENEWIZ. One microgram extracted RNA from each tissue (muscle, skin, heart, liver, brain, gill, eye, and kidney) was used to construct RNA libraries. Next generation sequencing library preparation was constructed according to the manufacturer’s protocol. The sequencing results were used to annotate the genome.

### Data filtering and genome size estimation

Illumina reads were trimmed with trim_galore v0.4.1 with default setting (http://www.bioinformatics.babraham.ac.uk/projects/trim_galore/, access by 2021/03/01) called by a custom Perl script trim_adaptor.pl (Yuan *et al.* 2019). Nanopore reads were kept if their average quality score was greater than 7 according to GrandOmics. Hi-C data were trimmed as trimming the regular Illumina reads. Trimmomatic v0.36 (Trimmomatic, RRID: SCR_011848) (Bolger *et al.* 2014) was used to trim transcriptome reads with default settings.

The trimmed Illumina reads were used to evaluate the genome size. The genome of *R. similis* was evaluated using Jellyfish v2.2.10 (Jellyfish, RRID: SCR_005491) (Marçais and Kingsford 2011) with the parameter “-m 21-s 1G” and K-mer set as 21. The frequency of K-mers with size of 21 was counted. Genomescope v1.0.0 (GenomeScope, RRID: SCR_017014) (Vurture *et al.* 2017) was used to estimate genome size, duplication and heterozygosity, with a maximum k-mer coverage rate of 16.6. The genome size was calculated by size = k-mer number/peak depth.

### *De novo* genome assembly

The nanopore data were assembled using NextDenovo (https://github.com/Nextomics/ NextDenovo, access by 2021/03/05) with the default parameters. Due to the high heterozygosity of the genome assembled with NextDenovo, purge_haplogs were used to remove redundancy after gene assembly (https://bitbucket.org/mroachawri/purge_haplotigs, access by 2021/03/11), the parameters of purge_haplogs with low, mid and high cutoff value were 10, 45, and 110 respectively. Illumina sequencing data were used to correct the NextDenovo assembly using NextPolish with default parameters (https://github.com/Nextomics/NextPolish, access by 2021/03/13). *The detail parameters for NextDenovo and NextPolish analyses can be found inSupplementaryFile*. The polished contigs were anchored onto chromosomes with Hi-C data using ALLHIC (https://github.com/tangerzhang/ALLHiC, access by 2021/03/18), while low-quality reads were removed using SAMTools (SAMTOOLS, RRID: SCR_002105) (Li *et al.* 2009). Genome assembly quality and gene integrity were evaluated using Benchmarking Universal Single-copy Orthologs v3.0.1 (BUSCO, RRID: SCR_015008) (Simão *et al.* 2015) with a database of 20 species called “vertebrata_odb10,” including 3354 vertebrate core genes. RepeatModeler v1.0.8 (RepeatModeler, RRID: SCR_015027) (Smit and Hubley 2010) with the default parameters was used to search for the repetitive sequences in the *R. similis* genome. RepeatMasker V4.0.7 (RepeatMasker, RRID: SCR_012954) (Smit *et al.* 2015) was used to mark the repeated sequences of the assembled genome according to the constructed repetitive sequence database. LTR elements in repeat sequences also were examined using LTR-FINDER with default parameters (Xu and Wang 2007).

### Transcriptome assembly

RNA-seq data from eight different tissues were assembled separately using the newly assembled genome as a reference with Trinity V2.11.0 (Trinity, RRID: SCR_013048) (Haas *et al.* 2013). Full-length transcripts of the variable splicing subtype were reported, and transcripts of the paralogous genes were distinguished. Finally, all transcripts from different tissues were combined, and the repetitive transcripts were removed using SeqKit (SeqKit, RRID: SCR_018926) (Shen *et al.* 2016). Quality of the transcriptome assembly was assessed using the same method as in genome assembly. The final transcripts were used for gene annotation.

### Gene prediction and annotation

Genes were predicted using Maker v2.31.10 pipeline (MAKER, RRID: SCR_005309) (Holt and Yandell 2011). All genes of the Gobiidae were retrieved from NCBI (https://www.ncbi.nlm.nih.gov/protein/? term=txid8220[Organism: exp], access by 2021/3/23), which was used as references for gene prediction. The transcriptome results also were used for annotation. The transcriptome was mapped to the genome using BWA (BWA, RRID: SCR_010910) (Li 2013). The hidden Markov models of Augustus (Augustus, RRID: SCR_008417) (Stanke and Waack 2003) and SNAP (Korf 2004) were trained by the resulting gene models for iterative genome annotation. The iteration was repeated twice. The Maker annotation was run three times (Holt and Yandell 2011). The same default settings were kept for the second and third runs, except that the maker_gff was replaced with the file generated in the previous step and the est_pass, altest_pass, protein_pass, and rm_pass were all changed to 1. The integrated gene set was first transformed into amino acid sequences, and then annotated by searching existing databases, including InterProScan5 (Jones *et al.* 2014), NCBI NR (NonRedundant Protein Sequence Database), and the KEGG (Kyoto Encyclopedia of Genes and Genomes) (Kanehisa and Goto 2000). BLASTP (BLASTP, RRID: SCR_001010) (Camacho *et al.* 2009) was used to search for known functional motifs, domains, and families in the InterProScan5 and NR databases to further annotate the predicted genes. BlAST2GO (Conesa *et al.* 2005) was used in KEGG analysis.

### Gene family analysis

Orthofinder (OrthoFinder, RRID: SCR_017118) (Emms and Kelly 2019) was used to analyze the orthologous genes and divide the orthologous groups. The protein sequences of 11 species (*Boleophthalmus pectinirostris, Cottoperca gobio, Cyclopterus lumpus, Danio rerio, Gasterosteus aculeatus, Oreochromis niloticus, Sphaeramia orbicularis, Sander lucioperca, Takifugu rubripes, Periophthalmus magnuspinnatus*, and *Rhinogobius similis*) were downloaded from NCBI (https://www.ncbi.nlm.nih.gov, access by 2021/3/23), and the annotation sequences of *Neogobius melanostomus* were retrieve from the zenodo database (https://zenodo.org/record/3561919/files/Supplemental_Material_S1_Round%20goby_Genome_Annotation.gz?download=1, access by 2021/10/23). Alternative splicing forms were removed, and only the longest transcript was retained to reduce redundancy. All-by-all BLAST search was performed on the above 12 groups of proteins. Then, a Markov clustering algorithm (OrthoMCL DB: Ortholog Groups of Protein Sequences, RRID: SCR_007839) (Fischer *et al.* 2011) was used to cluster the protein sequences. The single-copy orthologous genes were used to construct a maximal likelihood (ML) tree using the RAxML (RAxML, RRID: SCR_006086) (Stamatakis 2014). An ultrametric tree was obtained using r8s (r8s, RRID: SCR_021161) (Sanderson 2003) based on the resulting ML tree. The expansion and contraction of gene families were analyzed using CAFÉ (Computational Analysis of gene Family Evolution, RRID: SCR_018924) (Han *et al.* 2013), which estimates the birth-death (λ) parameter based on a given ultrametric tree and number of gene families.

### Analysis of population history of *Rhinogobius similis*

The historical population size of *R. similis* was inferred from diploid genome sequences using pairwise sequentially Markovian coalescent (PSMC, RRID: SCR_017229) (Li and Durbin 2011). The genome index of *R. similis* was constructed, and the bwa-mem algorithm of bwa v0.7.17 (Li 2013) was used to map the 43× paired-end fastq data (R1 and R2) to the genome of *R. similis*. SamTools v0.1.19 was used to generate the diploid consensus with default settings (https://github.com/lh3/psmc, access by 2021/03/18). The maturing age of the *R. similis* was set as one year, and the substitution rate was set as 2.5 × 10^−8^ per site per year (Liu *et al.* 2017) for PSMC analysis.

## Results and discussion

### Genome characteristics of *Rhinogobius similis*, genome assembly and evaluation

A total of 40.84 Gb (Coverage 43×) data were collected from Illumina resequencing, with 36.27 Gb paired-end reads kept and used for further analyses after removing low-quality reads (Supplementary Table S1). According to the single peak of k-mer (coverage = 16.6), the estimated genome size was 827.7 Mb (Supplementary Figure S1). The heterozygosity of the genome was 1.51%–1.66% (Supplementary Table S2). The low genetic diversity seen in *R. similis* probably is due to the founder effects, which was often seen in rapid invasive species (Mayr 1954).

A total of 66.96 Gb (coverage of 79×) of nanopore filtered data was used for de novo assembly, resulting in a genome of 902.41 Mb, close to that estimated by jellyfish, indicating that the assembling results are ideal. A nonredundant genome of 889.96 Mb was obtained after removing redundant sequences. The Illumina resequencing data were used to correct the nonredundant genome. A total of 1373 contigs were assembled, of which 262 contigs longer than 1 Mb accounting for 64.2% of the genome size. The Contig N50 was 1.54 Mb, with the longest one, 7.56 Mb. The genome GC content was 40.15% (Table 1). Using Hi-C data, 1373 contigs were anchored onto 22 pairs of chromosomes, leaving no remaining contigs unassembled and no contigs from other species (http://www.genomesize.com/, access by 2021/03/18) (Figure 1). The final genome assembly was 890.10 Mb and with a scaffold N50 of 41.51 Mb (Table 1). The BUSCO evaluation showed that 93.02% complete and 3.79% partial of the 3354 vertebrate BUSCO genes were captured (Supplementary Table S3).

**Table 1:** Statistics of NextDenovo genome assembly of *Rhinogobius similis*

Statistics	Values
Primary genome assembly	
Genome size (Mb)	902.41
Contigs	1373
Contig N50 (Mb)	1.54
>1 Mb contigs	262
>1 Mb contigs ratio	64.2%
Longest contig (Mb)	7.56
GC%	40.13%
Chromosome-level genome assembly	
Genome size (Mb)	890.10
Number of chromosomes	22
Scaffold N50 (Mb)	41.51
Longest contig (Mb)	53.7
GC%	40.15%

### Repetitive sequences, gene prediction, and annotation

Repetitive sequences accounted for 316.85 Mb (34.92%) of the genome at the chromosome level. The repetitive sequences included total interspersed repeats (31.80%), simple repeats (2.34%), and others. The total interspersed repeats can be further divided into retroelements (30.13%), DNA transposons (1.64%), simple repeats (2.34%), small RNA (0.05%), satellites (0.05%), low complexity (0.60%), and others (Supplementary Table S4).

A total of 31,089 protein-coding genes were found. The predicted protein-coding genes were compared with known databases, including Interproscan5, NCBI NR, and KEGG. Among them, 96.08% (similarity > 30%) genes were significantly similar to the known coding genes in the NCBI database, 86.50% (26,893) genes were annotated by using InterProScan5, and 36.62% (11,386) genes were annotated with KEGG pathway (Supplementary Table S5).

### Evolution of gene families

The phylogenetic tree of *R. similis* and 11 other fish species showed that *R. similis* was close to *B. pectinirostris* and *P. magnuspinnatus* (Figure 1). The differentiation time of *R. similis* from the mudskippers (*B. pectinirostris* and *P. magnuspinnatus*) was about 45.74 Mya. We selected the rapidly changing gene families of four species and constructed the Venn Graph, including *B. pectinirostris*, *N. melanostomus*, *P.magnuspinnatus*, and *R. similis* (Supplementary Figure S2). In *R. similis*, there were 1910 gene families expanded, 1171 gene families contracted and 170 gene families changed rapidly. In *B. pectinirostris*, 957 gene families expanded, 856 contracted, and 48 rapidly changed. In *N. melanostomus*, 2908 gene families expanded, 1885 contracted, and 260 rapidly changed. In *P. magnuspinnatus*, 278 gene families expanded, 862 contracted, and 46 rapidly changed. There only one gene family changed rapidly in all four species at the same time, expanding rapidly in *P. magnuspinnatus* and *R. similis* and contracting rapidly in *B. pectinirostris* and *N. melanostomus.* This family (*PF05970*) includes homologs of the PIF1 helicase, which inhibits telomerase activity and is cell cycle regulated. Telomerase activity affected the lifespan of zebrafish (Henriques *et al.* 2013). Telomerase mutant zebrafish have shorter telomeres. Among the four species, the longest life span of *R. similis* and *P. magnuspinnatu* is 4 years (Washio *et al.* 1991; Wu 2008), whereas life span of *B. pectinirostris* is seven years (Shi *et al.* 2014), and *N. melanostomus* is 6 years (Sokołowska and Fey 2011). Therefore, the rapidly expansion of *PF05970* gene family may be responsible for the short longevity of *R. similis*.

### Population history of *R*. *similis*

The effective population size (Ne) of *R. similis* varied in the range of 1.6–6.0 × 10^4^ from 400 to 10 Ka (Figure 1). The effective population size of *R. similis* experienced a drastic decline started from 400 to 100 Ka, which concurred with the Guxiang Glacial Stage. Shanghai is located in the lower reaches of the Yangtze River basin, and development of glaciers might reduce water flowing downstream, resulted in the decline of *R. similis* population. From 100 to 30 Ka, there were different glacial periods, including the Interglacial, Guxiang Glaciation, Last Interglacial (Cui *et al.* 2011), but the population size of *R. similis* maintained at about 1.6–2.0 × 10^4^. From 30 to 10 Ka, the population of *R. similis* gradually increased, which may be attributed to several warm periods during this time, and the influence of the Kuroshio.

## Conclusion

We assembled a high-quality chromosome-level genome of *R. similis*. As the first genome of the genus *Rhinogobius*, it may serve as a valuable resource for systematic study of the 100 described species of the *Rhinogobius* and many more undescribed ones. The data also can be useful in comparative studies focusing on molecular mechanisms of adaptive traits of the genus *Rhinogobius*.

## Data availability

The raw reads and RNA sequencing data have been uploaded in the SRA under Bioproject number PRJNA730052. The genome assembly files are under accession number of JAHMKA000000000. This whole-genome shotgun project has been deposited at DDBJ/ENA/GenBank under the accession JAHMKA000000000. The version described in this study is version JAHMKA010000000.

Supplementary material is available at *G3* online.

## Supplementary Material

jkab395_Supplementary_Data
