# Supplementary material for: A high-resolution genome of an euryhaline and eurythermal rhinogoby (Rhinogobius similis Gill 1895)
Source: G3 (Bethesda). 2021 Nov 18;12(2):jkab395. doi: 10.1093/g3journal/jkab395 (PMC9210307; doi:10.1093/g3journal/jkab395)
Supplement: jkab395_Supplementary_Data [file jkab395_supplementary_data.docx]

**Supplemental Material for Hu et al., 2021.**

**Table S1.** Sequencing data for genome assembly of *Rhinogobius similis*.

**Table S2.** Genome characteristics of *Rhinogobius similis* estimated using Genomescope Version 2.0.

**Table S3**. The completeness of the assembled genome of *Rhinogobius similis* evaluated using BUSCO.

**Table S4.** The composition and proportion of *Rhinogobius similis* repeat sequences.

**Table S5** Number of the annotated genes of *Rhinogobius similis* found in different databases.

**Figure S1.** Histogram of the 21-kmer depth distribution of the sequencing reads of *Rhinogobius similis* plotted in GenomeScope. The K-mer with a coverage of 16.6 has the largest number (excluding the K-mer with too low coverage), which was used to calculate the genome size.

**Figure S2.** Venn graph comparing rapidly changed genes of *Rhinogobius similis* with that of *Boleophthalmus pectinirostris, Neogobius melanostomus,* and *Periophthalmus magnuspinnatus*.

**Table S1** Sequencing data for genome assembly of *Rhinogobius similis*.

| Sequencing libraries | Raw data (bp) | Clean data (bp) | Sequence coverage (×) |
| --- | --- | --- | --- |
| Illumina reads | 40,835,378,100 | 36,270,682,691 | 43 |
| Nanopore reads | 75,688,200,395 | 66,957,655,456 | 79 |
| Hi-C reads | 108,650,995,800 | 85,411,149,600 | 101 |
| Transcriptome reads | 58,413,937,800 | 54,782,376,550 | 65 |
| Total | 283,588,512,095 | 243,421,864,297 | 287 |

**Table S2.** Genome characteristics of *Rhinogobius similis* estimated using Genomescope Version 2.0.

| Characteristics | Min | Max |
| --- | --- | --- |
| Homozygous | 98.34% | 98.49% |
| Heterozygous | 1.51% | 1.66% |
| Genome haploid length | 817,514,908 bp | 827,750,672 bp |
| Genome repeat length | 262,808,047 bp | 266,098,558 bp |
| Genome unique length | 554,706,860 bp | 561,652,114 bp |
| Model fit | 74.01% | 98.94% |
| Read error rate | 0.71% | 0.71% |

**Table S3** The completeness of the assembled genome of *Rhinogobius similis* evaluated using BUSCO.

|  | Proteins | Percentage (%) |
| --- | --- | --- |
| Complete BUSCOs | 3,120 | 93.02 |
| Complete and single-copy BUSCOs | 3,099 | 92.40 |
| Complete and duplicated BUSCOs | 21 | 0.63 |
| Fragmented BUSCOs | 107 | 3.19 |
| Missing BUSCOs | 127 | 3.79 |
| Total BUSCO groups searched | 3,354 | 100 |

**Table S4.** The composition and proportion of *Rhinogobius similis* repeat sequences.

| Category | Number of elements | Length (bp) | Percentage |
| --- | --- | --- | --- |
| Retroelements | 193,894 | 274,244,894 | 30.13% |
| SINEs | 11,953 | 1,380,772 | 0.16% |
| Penelope | 1,067 | 163,270 | 0.02% |
| LINEs | 66,740 | 24,788,392 | 2.78% |
| CRE/SLACS | 0 | 0 | 0.00% |
| L2/CR1/Rex | 47,968 | 15,347,409 | 1.72% |
| R1/LOA/Jockey | 797 | 316,005 | 0.04% |
| R2/R4/NeSL | 125 | 30,371 | 0.00% |
| RTE/Bov-B | 8,780 | 5,679,186 | 0.64% |
| L1/CIN4 | 6,700 | 2,719,468 | 0.31% |
| LTR elements | 114,134 | 247,912,460 | 27.17% |
| BEL/Pao | 961 | 1,436,968 | 0.16% |
| Ty1/Copia | 218 | 1,422,959 | 0.16% |
| Gypsy/DIRS1 | 18,269 | 48,909,909 | 5.49% |
| Retroviral | 5,523 | 622,266 | 0.07% |
| unknown | 73,670 | 190,941,203 | 21.29% |
| DNA transposons | 155,748 | 14,593,054 | 1.64% |
| hobo-Activator | 90,969 | 7,935,329 | 0.89% |
| Tc1-IS630-Pogo | 13,774 | 1,975,873 | 0.22% |
| En-Spm | 0 | 0 | 0.00% |
| MuDR-IS905 | 0 | 0 | 0.00% |
| PiggyBac | 171 | 32,772 | 0.00% |
| Tourist/Harbinger | 2,257 | 212,329 | 0.02% |
| Other (Mirage, P-element, Transib) | 305 | 42,900 | 0.00% |
|  |  |  |  |
| Rolling-circles | 3,123 | 547,416 | 0.06% |
|  |  |  |  |
| Unclassified | 850 | 407,688 | 0.05% |
|  |  |  |  |
| Total interspersed repeats | 349,425 | 246,475,492 | 31.80% |
|  |  |  |  |
| Small RNA | 4,775 | 451,583 | 0.05% |
|  |  |  |  |
| Satellites | 1,095 | 408,899 | 0.05% |
| Simple repeats | 296,068 | 20,817,740 | 2.34% |
| Low complexity | 50,449 | 5,380,566 | 0.60% |

**Table S5** Number of the annotated genes of *Rhinogobius similis* found in different databases.

| Database | Number of genes | Percentage (%) |
| --- | --- | --- |
| InterProscan5 | 26,893 | 86.50 |
| NR (similarity >30%) | 29,871 | 96.08 |
| KEGG | 11,386 | 36.62 |
| GO | 5,943 | 19.12 |
| PANTHER | 5,812 | 18.69 |
| SUPERFAMILY | 2,678 | 8.61 |
| Pfam | 2,435 | 7.83 |
| Gene3D | 2,695 | 8.67 |
| ProSiteProfiles | 1,785 | 5.74 |
| SMART | 1,396 | 4.49 |
| CDD | 651 | 2.09 |
| MobiDBLite | 6,634 | 21.34 |
| ProSitePatterns | 549 | 1.77 |
| PIRSF | 92 | 0.29 |
| Coils | 1,360 | 4.37 |
| PRINTS | 615 | 1.98 |
| TIGRFAM | 148 | 0.48 |
| SFLD | 16 | 0.05 |
| Hamap | 27 | 0.08 |


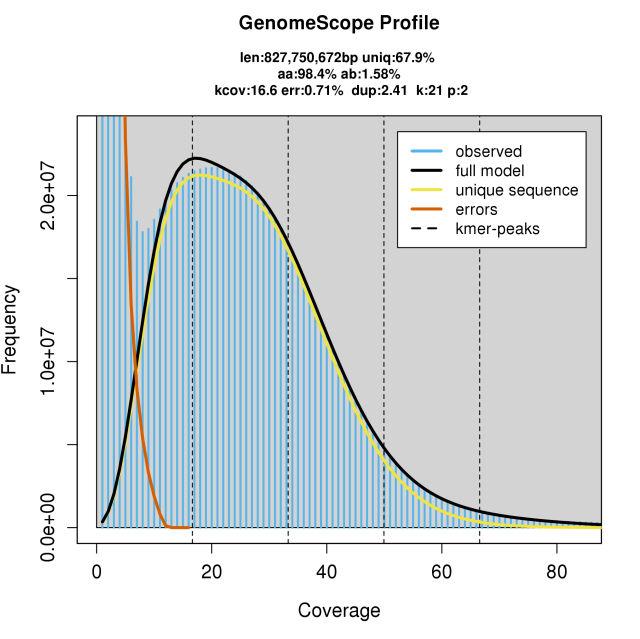


**Figure S1.** Histogram of the 21-kmer depth distribution of the sequencing reads of *Rhinogobius similis* plotted in GenomeScope. The K-mer with a coverage of 16.6 has the largest number (excluding the K-mer with too low coverage), which was used to calculate the genome size.


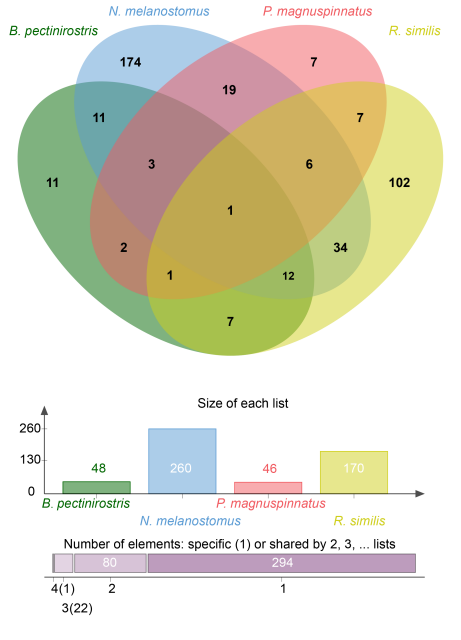


**Figure S2.** Venn graph comparing rapidly changed genes of *Rhinogobius similis* with that of *Boleophthalmus pectinirostris, Neogobius melanostomus,* and *Periophthalmus magnuspinnatus*.

Custom scripts:

1) Calculate genome size, N50 and GC content

import sys

inputPath = ''

for i in range(len(sys.argv)):

if sys.argv[i] == '-i':

inputPath = sys.argv[i + 1]

alllen = 0

seqname = ''

seqDict = {}

filelist = open(inputPath).readlines()

countN = 0

countGC = 0

for line in filelist:

if line[0] == '>':

seqname = line

seqDict[seqname] = 0

else:

seqDict[seqname] += len(line) - 1

countN += line.count('N')

countGC += line.count('G') + line.count('g') + line.count('C') + line.count('c')

lenList = []

for seq in seqDict:

alllen += seqDict[seq]

lenList.append(seqDict[seq])

lenList.sort(reverse=True)

N50 = 0

stat = 0

for l in lenList:

stat += l

if stat >= alllen * 0.5:

N50 = l

break

print('All len: ', alllen)

print('N50: ', N50)

print('GC%: ', (float(countGC) / alllen) * 100, '%')

print('N%: ', (float(countN) / alllen) * 100, '%')

**Nextdenovo parameter**

[General]

job_type = local # local, slurm, sge, pbs, lsf

job_prefix = CL2600

task = all # all, correct, assemble

rewrite = yes # yes/no

deltmp = yes

rerun = 3

parallel_jobs = 5 # number of tasks used to run in parallel

input_type = raw

input_fofn = input.fofn

workdir = 01_rundir

[correct_option]

read_cutoff = 1k

seed_cutoff = 18231 # minimum seed length

blocksize = 2g

pa_correction = 3 # number of corrected tasks used to run in parallel, overwrite ${parallel_jobs} only for this step

seed_cutfiles = 4

sort_options = -m 20g -t 10 -k 40 # -k, max depth of each overlap, should <= average sequencing depth

minimap2_options_raw = -x ava-ont -t 10 # change to ava-pb for PacBio CLR data

correction_options = -p 10

[assemble_option]

minimap2_options_cns = -x ava-ont -t 10 -k17 -w17 # change to ava-pb for PacBio CLR data

nextgraph_options = -a 1

**nextpolish parameter:**

[General]

job_type = local

job_prefix = nextPolish

task = default

rewrite = yes

rerun = 3

parallel_jobs = 7

multithread_jobs = 9

genome = /mnt/genome1/Lab_Users/LL/cp_genome/coilia/0_raw_data/genome/2552_1_CM_hic/1_nano_assembly/2553_2/6-bridge_contigs/polished_contigs.fasta

genome_size = auto

workdir = /mnt/genome1/Lab_Users/LL/cp_genome/coilia/0_raw_data/genome/2552_1_CM_hic/2_polish

polish_options = -p {multithread_jobs}

[sgs_option]

sgs_fofn = /mnt/genome1/Lab_Users/LL/cp_genome/coilia/0_raw_data/genome/2552_1_CM_hic/2_polish/ngs_fq_path.txt

sgs_options = -max_depth 100

lgs_minimap2_options = -x map-ont
